# The test characteristics of a biased or ignorant diagnostician

**DOI:** 10.1186/s12911-022-01950-2

**Published:** 2022-08-09

**Authors:** Amnon Sonnenberg

**Affiliations:** Division of Gastroenterology/Hepatology, Oregon Health & Science University, Portland VA Medical Center P3-GI, 3710 SW US Veterans Hospital Road, Portland, OR 97239 USA

**Keywords:** Bayes formula, Bias, Decision making, Dunning–Kruger effect, Judgement, Test characteristics

## Abstract

**Background:**

A human diagnostician may harbour a special bias towards favourable positive or negative test results. The aim of the present analysis is to describe in quantitative terms how bias can affect the test characteristics of a human tester.

**Methods:**

Whereas an unbiased tester would give absolute (100%) preference to true positive or true negative test results, and no (0%) preference to any false positive or false negative test results, a biased tester may harbour some preferences towards false positive or false negative tests. Such bias can be phrased in terms of a separate sensitivity–specificity matrix. The bias matrix multiplied with the original test matrix yields the biased test matrix. Similarly, the extent of ignorance by a human tester about the diagnosis is modelled as a separate sensitivity–specificity matrix, which captures the concordance between positive and negative diagnoses made by an ignorant and expert diagnostician.

**Results:**

Increasing bias or ignorance result in decreasing test performance with decreasing positive predictive values until the test completely loses its discriminatory power. With more pronounced bias towards false test results, any positive test outcome may even become misinterpreted as predicting the non-existence of a given diagnosis.

**Conclusions:**

The proposed model helps to understand in quantitative terms, how bias and ignorance can alter a diagnostician’s interpretation of test outcomes and result in diagnostic errors.

## Introduction

The performance of a medical test is described in terms of its test characteristics [1–4]. Like the performance of any other test, the function of a human diagnostician, performing a physical exam, taking a history, or using any type of diagnostic instrument, can also be captured in terms of such test characteristics [5]. Any dichotomous judgement by a decision maker lends itself to be phrased in terms of its sensitivity and specificity. A good test is associated with high sensitivity and specificity values. The quality of any test can become compromised by bias or ignorance. A human diagnostician may harbour a special bias towards favourable positive or negative test results. Such bias may stem from financial gains associated with a positive diagnosis, a personal interest in establishing an interesting diagnosis, or trying to avoid the diagnosis of an unpleasant or dangerous disease. Similar to bias, ignorance by the tester about the true nature of the diagnosis tested for can also affect the test outcome. The aim of the present analysis is to describe in quantitative terms how bias and ignorance influence the test characteristics of a human tester.

## Methods

Throughout the article, T+ and T– represent a positive or negative test result, respectively. Dx+ and Dx– represent a positive or negative diagnosis, respectively. In terms of probability theory, the fraction (or probability, Pr) of true positive (TP) test results, given a positive diagnosis, is defined as

1 $${\text{TP}} = {{\text{Pr}}} ({\text{T}}+| {\text{Dx}}+).$$

Similarly, the fraction of false positive (FP) test results, given a negative diagnosis, is defined as

2 $${\text{FP}} = {{\text{Pr}}} ({\text{T}}+| {\text{Dx}}-).$$

The two fractions of false negative (FN) and true negative (TN) test results are defined as

3 $${\text{FN}} = {{\text{Pr}}} ({\text{T}}-| {\text{Dx}}+)$$

and

4 $${\text{TN}} = {{\text{Pr}}} ({\text{T}}-| {\text{Dx}}-).$$

In the medical literature, the fractions of true positive and true negative test results are also called sensitivity and specificity, respectively [1-4]. The sum of true positive and false negative test fractions, as well as the sum of false positive and true negative test fractions, both add up to 100%. The four fractions are generally arranged in form of a two-by-two matrix as shown in the tables of the present article.

Sequential testing applies to situations when the output of a prior test provides the input for a later test and becomes modified or updated by a subsequent test [5–7]. Based on its appearance, for instance, a lesion detected by endoscopy or other imaging techniques may be associated with high sensitivity and specificity, but to really establish the diagnosis a physician must first be able to visualize the pathognomonic sign and then also correctly interpret its meaning. The overall sensitivity and specificity, therefore, depend on the test characteristics of the image itself, as well as the test characteristics of the physician in being able to elicit and interpret the diagnostic finding. In other clinical scenarios, test information flows from a specialist, such as radiologist, gastroenterologist, pulmonologist, etc., to a general practitioner. Within such chain of interacting physicians, the overall test performance becomes modified by the ability of each individual physician to understand and correctly interpret the information that passes through. A journal editor who relies in her own decision to accept a manuscript on prior assessment by a reviewer also acts a sequential tester. As a final example, consider a junior physician, such as resident or fellow who learns from a senior attending physician. At least in the beginning of their career, the overall test performance by the junior physicians relies on their knowledge, skills and ability in adopting the teacher’s own (potentially limited) performance [8].

The joint influence of two consecutive tests corresponds to the multiplication of their respective test matrices [5, 6]. According to the rules of matrix algebra, multiplying each row from the secondary test matrix with each column from the primary test matrix yields the elements of their combined matrix, which are located at row-column intersection [9]. The overall fraction of true positives (TPs) following two sequential tests corresponds to:

5 $${\text{TP}}_{{\text{s}}} = {\text{TP}}_{2} \cdot {\text{TP}}_{1} + {\text{FP}}_{2} \cdot {\text{FN}}_{1} .$$

Similarly, the overall fraction of false positives (FNs)following two sequential tests corresponds to

6 $${\text{FP}}_{{\text{s}}} = {\text{TP}}_{2} \cdot {\text{FP}}_{1} + {\text{FP}}_{2} \cdot {\text{TN}}_{1}$$

Lastly, the overall fractions of false negatives (FNs) and true negatives (TNs) correspond to

7 $${\text{FNs}} = {\text{FN}}_{2} \cdot {\text{TP}}_{1} + {\text{TN}}_{2} \cdot {\text{FN}}_{1}$$

and

8 $${\text{TNs}} = {\text{FN}}_{2} \cdot {\text{FP}}_{1} + {\text{TN}}_{2} \cdot {\text{TN}}_{1}.$$

Such calculations can be easily performed on an Excel spreadsheet (from Microsoft, Redmond, WA), using its built-in MMULT array function [10].

For the purpose of the present analysis, the influence of bias or ignorance on the test matrix is modelled as a quasi add-on sequential test “colouring” the baseline test characteristics. A human tester may harbour different preferences regarding the four combinations of positive or negative test results in the presence or absence of a diagnosis. The bias of a human tester is reflected by a separate sensitivity–specificity matrix.

An unbiased tester would give absolute (100%) preference to true positive or true negative test results, and no (0%) preference to any false positive or false negative test results. A bias towards a positive test outcome would increase the fraction of false positive tests at the expense of true negative testes. Such bias characterizes instances where the diagnostician is overly keen in finding an explanation for the patient’s symptoms at the expense of her professional objectivity or has a professional interest in establishing a diagnosis that is interesting or provides a financial advantage to the physician herself. A bias towards a negative test outcome would increase the fraction of false negative tests at the expense of true positive testes. Such bias characterizes instances where the diagnostician is concerned about finding a diagnosis, which would entail grave and unpleasant consequences for the patient, or which exceeds the physician’s professional means of managing it. Lastly, a physician’s attitude may be characterized by a combination of both, a positive and negative bias towards the primary test results. For instance, out of financial or other professional interests, a radiologist may ignore the findings of abdominal ultrasound and recommend other more expensive means of radiographic imaging. A gastroenterologist may generally mistrust intestinal diagnoses made by abdominal CT scans or by other endoscopists than herself. Other such examples abound.

The influence of bias on the overall test performance is represented by the biased test matrix, which corresponds to the product of the bias matrix with the test matrix. Similarly, the extent of ignorance by a human tester about the diagnosis is modelled as a separate sensitivity–specificity matrix, which captures the concordance between positive and negative diagnoses made by an ignorant and expert diagnostician.

Bayes’ formula is used to calculate the positive predictive value of a positive test, that is, the change from pre-test to post-test probability of a specific disease [1–4].

9 $$PPV = \frac {p \cdot TP} {p \cdot TP + (1-p) \cdot FP}$$

The pre-test probably of a given disease (p) is defined as the ratio of individuals in whom the disease occurs and the population size tested. (It could also represent a known prevalence of the disease or just an estimate of its a-priori probability of its occurrence.) The influence of multiple consecutive separate tests is calculated by repetitive applications of Bayes’ formula. For the purpose of the present analysis, the same test characteristics are assumed to apply to consecutive tests.

## Results

Table 1 contains several scenarios of two sequential tests, with the second test depending on the test performance of the first test. The combined test characteristics correspond with the multiplication of the two test matrices. The first scenario illustrates that a perfect 2nd tester with both sensitivity and specificity values of 100% would leave the test characteristics of the primary test unaffected. The second scenario illustrates a situation where in an imperfect 2nd tester interprets or builds on the results of a prefect primary test. Such situations arise, for instance, if the results obtained by using a highly sensitive and specific instrument depend on the manual or cognitive skills of its less than perfect operator. In the majority of cases, sequential testing leads to a deterioration of the overall test characteristics. This phenomenon is illustrated by the third scenario of Table 1 where the 1st and 2nd test both yield imperfect test results. Such scenario would, for instance, apply to a situation, where an attending physician would assess a clinical status based on the report by a medial resident. Their combined test performance turns out worse than the test performance of each individual tester alone. A completely worthless secondary test is characterized by a test matrix with sensitivity and specificity values of 50%. Any secondary test or tester with such characteristics would turn even the best test into worthless information. As all examples of Table 1 illustrate, the overall (combined) outcome of two sequential tests is at best only as good as the test performance of the lesser of the two tests. In other words, a tester cannot appreciate any extraneous test results beyond his/her very own limitations or level of understanding.

**Table 1:** Examples of the joint influence of two sequential tests

Scenario	2nd Test matrix		1st Test matrix		Combined test matrix	
	Dx + (%)	Dx− (%)	Dx + (%)	Dx− (%)	Dx + (%)	Dx− (%)
*Perfect 2nd tester*						
T+	100	0	80	10	80	10
T−	0	100	20	90	20	90
*Perfect 1st tester*						
T+	80	10	100	0	80	10
T−	20	90	0	100	20	90
*Imperfect 2 testers*						
T+	80	10	70	15	59	20
T−	20	90	30	85	41	80
*Ignorant 2nd tester*						
T+	50	50	70	15	50	50
T−	50	50	30	85	50	50
*Ignorant 2nd tester*						
T+	50	50	100	0	50	50
T−	50	50	0	100	50	50

T+ and T− represent a positive or negative test result, respectively. Dx+and Dx− represent a positive or negative diagnosis, respectively

The influence of bias harboured by a human tester lends itself to be phrased in terms of a sequential secondary test, with the potential to impugn the characteristics of the primary test. Table 2 provides seven examples for the influence of bias on a test characterized by sensitivity and specificity values of 80% and 90%, respectively, with each consecutive example downward representing an increasingly more pronounced form of bias. Without bias, as shown by the top example, the test matrix remains unaffected, and a positive test result increases the disease probability from 20 to 67%. An increasing bias results in decreasing test performance with decreasing positive predictive values until the test completely loses its discriminatory power in the fourth example. With even more pronounced bias towards false test results, any positive test outcome becomes erroneously interpreted as predicting the non-existence of the diagnosis tested for.

**Table 2:** Seven examples of the same test matrix affected by bias of increasing magnitude

	Bias		Test matrix		Biased test matrix			p and PPV of diagnosis (%)	p and PPV of alternative diagnosis (%)
	Dx+ (%)	Dx− (%)	Dx+ (%)	Dx− (%)	Dx+ (%)	Dx− (%)			
1st Example									
T+	100	0	80	10	80	10	p	20	80
T−	0	100	20	90	20	90	PPV	67	33
2nd Example									
T+	80	20	80	10	68	26	p	20	80
T−	20	80	20	90	32	74	PPV	40	60
3rd Example									
T+	60	40	80	10	56	42	p	20	80
T−	40	60	20	90	44	58	PPV	25	75
4th Example									
T+	50	50	80	10	50	50	p	20	80
T−	50	50	20	90	50	50	PPV	20	80
5th Example									
T+	40	60	80	10	44	58	p	20	80
T−	60	40	20	90	56	42	PPV	16	84
6th Example									
T+	20	80	80	10	32	74	p	20	80
T−	80	20	20	90	68	26	PPV	10	90
7th Example									
T+	0	100	80	10	20	90	p	20	80
T−	100	0	20	90	80	10	PPV	5	95

T+ and T− represent a positive or negative test result, respectively; Dx+and Dx− represent a positive or negative diagnosis, respectively; p & PPV represent pre-test probability & positive predictive value, respectively

In general, the sensitivity and specificity of a test cannot drop below 50%. However, this rule does not apply to a biased human tester. Table 3 serves to illustrate how a strong bias can completely revert the meaning of a positive test outcome into its opposite. The top example refers to a completely unbiased tester whose inexistent bias leaves the test matrix (and the test outcome) unaffected. Assuming a 50/50 split in the pre-test probability for the presence of a diagnosis or its alternative, the unbiased evaluation of a positive test outcome increases the diagnostic probability from its pre-test value of *p* = 50% to a post-test value of PPV = 94%. As shown by the second example of Table 3, in a strongly biased tester, however, the same positive test outcome becomes interpreted completely differently by assigning the *alternative* (negative) diagnosis a falsely high positive predictive value of PPV = 83%. By inverting the performance characteristics of the test matrix, a strong bias may completely invalidate the test outcome and lead to a seeming confirmation the tester’s biased perception.

**Table 3:** Examples for the influence of bias and ignorance on test outcomes

Scenario	Bias or ignorance			Test matrix			Biased test matrix			Outcome	
	Dx+ (%)	Dx− (%)		Dx+ (%)	Dx− (%)		Dx+ (%)	Dx− (%)		Dx+ (%)	Dx− (%)
*Unbiased tester*											
Dx+	100	0	T+	80	5	T+	80	5	p	50	50
Dx−	0	100	T−	20	95	T−	20	95	PPV	94	6
*Biased tester*											
Dx+	0	100	T+	80	5	T+	20	95	p	50	50
Dx−	100	0	T−	20	95	T−	80	5	PPV	17	83

	Expert Dx+ (%)	Expert Dx− (%)		Dx+ (%)	Dx− (%)		Dx+ (%)	Dx− (%)		Dx+ (%)	Dx− (%)
*Informed tester*											
Amateur Dx+	100	0	T+	80	5	T+	80	5	p	50	50
Amateur Dx−	0	100	T−	20	95	T−	20	95	PPV	94	6
*Ignorant tester*											
Amateur Dx+	60	30	T+	80	5	T+	54	32	p	50	50
Amateur Dx−	40	70	T-	20	95	T-	46	69	PPV	63	37

T+ and T− represent a positive or negative test result, respectively; Dx+ and Dx− represent a positive or negative diagnosis, respectively; p & PPV represent pre-test probability & positive predictive value

**Fig. 1:**
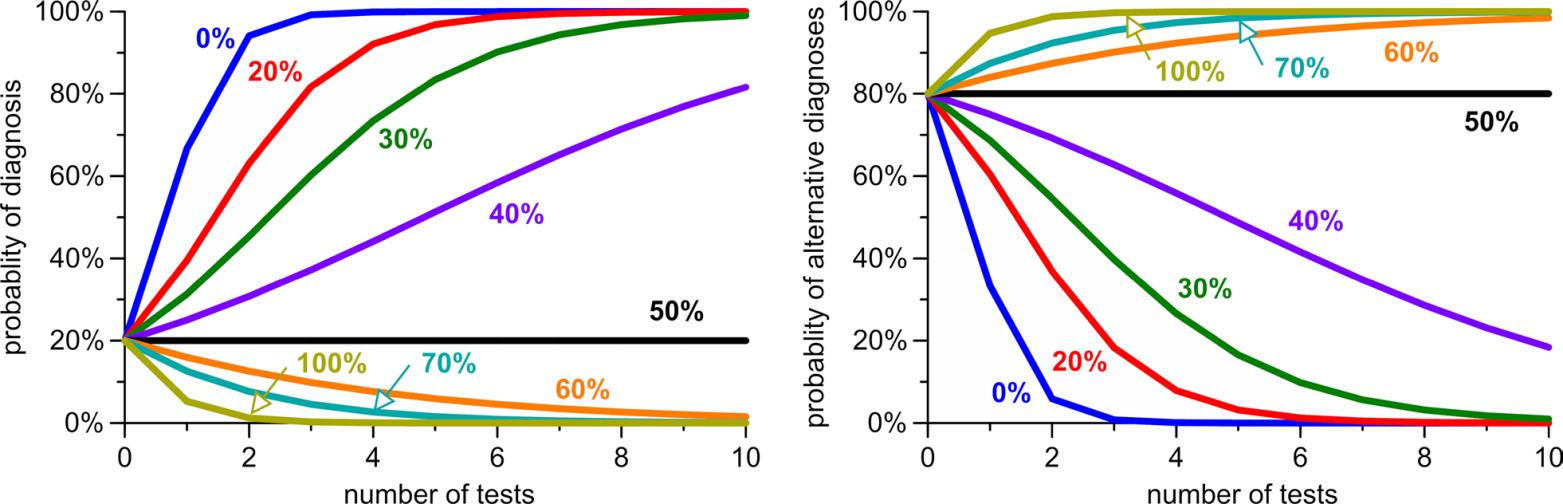
Influence of bias on test outcome. The positive predictive value of a diagnosis (left) and its alternatives (right) rise or fall with multiple consecutive tests. Each curve represents a bias of different magnitude, ranging from 0 to 100%

Figure 1 shows the impact of using multiple biased tests consecutively. Using unbiased test example from Table 2, it takes only 2 positive tests results to raise a pre-test probability from 20% to a post-test probability over 90%. With a 20% bias of favouring false tests, it takes 4 positive tests to raise the post-test probability over 90%, and with a 30% bias it would take more than 6 positive tests. A 50% bias renders the chain of consecutive tests worthless and completely unable to raise the diagnostic probability. Even more pronounced forms of bias would lead the diagnostician to misinterpret one or few positive tests as predicting the absence of the true diagnosis. This point is also illustrated by the right panel of Fig. 1, which shows the changes in the positive predictive values of alternative diagnoses associated multiple positive tests. If a particular alternative diagnosis were assigned a high pre-test probability, it would take only one or two biased tests to erroneously raise its post-test probability above 90% and seemingly prove its existence.

It is generally assumed that the diagnosis, whose presence or absence is being tested for, represents a clear-cut nosologic entity. However, sometimes the tester knows the diagnosis only by name without a detailed appreciation for its true appearance or consequences. There is a difference between knowing the terms and having a full grasp of the complex and multifaceted nature that is encapsulated by a short medical term. The difference between a fully understood diagnosis and superficial fluency with the medical term relates to the difference between an amateur and an expert. The concordance between positive and negative diagnoses made by an amateur and expert diagnostician can again be phrased in terms of a two-by-two sensitivity–specificity matrix (Table 3, third and fourth example). An informed tester will show a close (to 100%) concordance with any other expert diagnostician and leave the function of the test-matrix uncompromised. Compared with an expert, an ignorant tester will understand the true meaning of a diagnosis only in a fraction of instances. Ultimately, ignorance acts on the test matrix in a similar fashion as a bias, with the overall performance of the test being reflected by the product of the ignorance-matrix with the test-matrix. Again, such multiplication invariably results in a deterioration of the test characteristics with an overall reduction in test performance.

## Discussion

It stands to reason that personal preferences would bias one’s perception and interpretation of any test outcome. Physicians like other professionals sometimes see the world less as it is and more as they would like it to appear. The present analysis tries to establish a quantitative relationship between the magnitude of such personal preferences and their effect on cognitive decline. These preferences are phrased in terms of a two-by-two matrix, which acts on the primary diagnostic test and leads to deterioration of its test characteristics. Ignorance exerts a similar effect as bias on the test-matrix. Although bias and ignorance constitute two different phenomena, ultimately, they both effect the test matrix in a similar fashion and reduce its overall efficacy.

Like any other type of diagnostic test, cognition and judgement by a human tester can also be described in terms of sensitivity and specificity values. Good judgement is characterized by high sensitivity and specificity values in recognizing abstract concepts or selecting between competing alternatives. The performance of human testers is a-priori limited by their very own levels of sensitivity and specificity in interpreting test outcomes or any given array of facts. An adage of hermeneutics states that humans only see what they know [11–13]. Accordingly, human testers cannot measure beyond their own level of competence or understanding, as reflected by their corresponding sensitivity and specificity values in assessing test outcomes. These levels of understanding may be determined by innate talents, intellectual aptitude, as well as bias and ignorance.

Test characteristics are usually accepted as given, but rarely questioned with respect to their function in the hands of a biased or ignorant human tester. The influence of bias and ignorance can markedly reduce the quality of a test as evidenced by its test characteristics. Although the focus of the present article is centred on the test performance of a physician, who interprets signs and symptoms in making a medical diagnosis, similar arguments apply to other human testers deciding between any types of dichotomous judgement options. Phrasing judgement in terms of a sensitivity–specificity matrix provides a useful framework to conceptualize the influence of bias and ignorance on decision making. The underlying mathematics involves little more than matrix multiplication, which is easy to execute and renders the outcome of calculations transparent for future reference. Despite its simplicity, the model provides valuable insights about the impact of two such relevant confounders, such as bias and ignorance, on human judgement.

Several caveats and limitations pertaining to the present analysis deserve to be mentioned. Among statisticians and epidemiologists, innumerable forms of bias are known to affect the accumulation and analysis of research data [14–16]. The present analysis is not meant to cover all types of different bias, such as selection bias, lead time bias, or even other forms of cognitive bias. It also does not consider bias with respect to the expected outcomes that would be expressed as monetary costs or in terms of effectiveness. The present analysis is restricted to the type of bias that could be expressed as personal preference for identifiable test outcomes. Besides the test characteristics, however, a diagnostician may also be *a-priori* biased in assigning false pre-test probabilities to various diagnostic alternatives. Such bias would further aggravate the impact of the bias illustrated in Table 2. In the examples shown, consecutive tests were assumed to carry the same test characteristics, and a bias of the same magnitude was chosen for false positive, as well as false negative test results, acting similarly on consecutive tests. These assumptions were made to simplify the calculations and make the results more transparent, but they do not affect the general applicability of the model. In reality, different tests would harbour varying sensitivity and specificity values, and different tests would also be affected by bias of varying magnitude.

In conclusion, the proposed model helps to understand in quantitative terms, how bias and ignorance can alter a diagnostician’s interpretation of test outcomes and result in errors of judgement or decision making. Even with its potential limitations in mind, the model might be insightful and applicable to different scenarios of decision analysis.

## Data Availability

All data generated or analysed during this study are included in this published article.
